# *DKC1* overexpression associated with prostate cancer progression

**DOI:** 10.1038/sj.bjc.6605299

**Published:** 2009-09-15

**Authors:** P Sieron, C Hader, J Hatina, R Engers, A Wlazlinski, M Müller, W A Schulz

**Affiliations:** 1 Urologische Klinik, Heinrich Heine Universität, Moorenstr. 5, Düsseldorf 40225, Germany; 2 Institut für Pathologie, Heinrich Heine Universität, Moorenstr. 5, Düsseldorf 40225, Germany

**Keywords:** prostate adenocarcinoma, dyskerin, siRNA, telomerase, apoptosis, tumour progression

## Abstract

**Background:**

Dyskerin encoded by the *DKC1* gene is a predominantly nucleolar protein essential for the formation of pseudouridine in RNA and the telomerase RNA subunit hTR. Inherited mutations inactivating dyskerin cause dyskeratosis congenita, a syndrome with progeroid features characterised by skin defects and haematopoiesis failure, as well as cancer susceptibility. In this study, we report *DKC1* overexpression in prostate cancers.

**Methods:**

Expression of *DKC1* was measured by quantitative RT–PCR in prostate cancer tissues in relation to hTR and the proliferation marker MKI67. Effects of dyskerin downregulation on proliferation, apoptosis and senescence of prostate cancer cell lines were determined.

**Results:**

*DKC1* was significantly overexpressed in prostate cancers, particularly in high-stage and recurring cases, correlating moderately with *hTR* and *MKI67*. Dyskerin downregulation in prostate carcinoma cell lines by siRNA diminished cell proliferation, but elicited neither apoptosis nor senescence. Apoptosis induction by TNF-*α* or tunicamycin was not enhanced. Long-term downregulation led predominantly to cell shrinking and loss of adhesion.

**Interpretation:**

*DKC1* upregulation in prostate cancers is common and likely to be necessary for extensive tumour growth. The phenotype of prostate carcinoma cell lines after dyskerin downregulation suggests that its most critical function is sustaining protein biosynthesis. Intriguingly, compromised function and overexpression of dyskerin can both contribute to cancer development.

Dyskerin, also known as Cbf5, is a predominantly nucleolar protein encoded by the *DKC1* gene at Xq28 (Mason *et al*, 2005; Kirwan and Dokal, 2008). As part of a snoRNA ribonucleoprotein complex, dyskerin catalyses the formation of pseudouridine in ribosomal and certain small RNAs (Filipowicz and Pogacic, 2002). Moreover, dyskerin is necessary for the biogenesis of hTR, the RNA component of telomerase, which is transcribed from the *TERC* gene. The hTR RNA contains a specific recognition motif for dyskerin, and dyskerin is retained in the core telomerase complex (Chang *et al*, 2002; Cohen *et al*, 2007). Very recently, dyskerin was reported to exert an additional function by regulating the translation of a number of anti-apoptotic proteins (Yoon *et al*, 2006).

Inherited mutations in *DKC1* cause the human hereditary syndrome, dyskeratosis congenita (Mason *et al*, 2005; Kirwan and Dokal, 2008). Owing to the localisation of *DKC1* at Xq28, males are predominantly affected, whereas females heterozygous for a mutant gene seem to compensate by selection of cells with the active functional allele. The symptoms of the disease are variable. In most cases, dyskeratosis congenita manifests initially as defects in reticulate skin pigmentation, nail dystrophy, and mucosal leukoplakia. Progressively deficient haematopoiesis is the main cause of death. Evidently, insufficient dyskerin function primarily affects tissues with rapid cell turnover, such as the skin and the haematopoietic system, likely as a consequence of impaired cell proliferation. Somewhat paradoxically, dyskeratosis congenita patients are also prone to develop cancers, particularly skin cancers and leukaemias. Moreover, in sporadic chronic lymphocytic leukaemia, *DKC1* expression is diminished, together with that of other telomerase-associated factors (Poncet *et al*, 2008). It is debated as to loss of which function of dyskerin is most crucial for the symptoms of dyskeratosis congenita. Some experts argue that insufficient protein synthesis resulting from defects in ribosome biogenesis may compromise tissue regeneration, whereas others emphasise that telomerase dysfunction caused by hTR deficiency may limit the renewal of tissue stem cells (Mason *et al*, 2005; Wong and Collins, 2006; Kirwan and Dokal, 2008). The latter argument is supported by the observation that inherited mutations in *TERC* elicit a similar, although generally milder form of the disease. Perhaps because of the defects in stem cell function, dyskeratosis congenita has some similarities to progeroid syndromes (Kirwan and Dokal, 2008).

Although increased nucleolar activity is a long-established indicator of malignancy (reviewed in Montanaro *et al* (2008b)), dyskerin expression and function in sporadic cancers have hardly been investigated. A pioneer study has reported dyskerin expression to be increased in several human cancer types, especially in breast cancers (Montanaro *et al*, 2006). In accord with the known biological functions of the protein, breast cancers with low dyskerin expression contained lower levels of pseudouridine and telomerase RNA than those with high expression. Moreover, cancers with high expression generally exhibited worse histopathological features and prognosis. In line with these findings, a microarray study from our group (Schulz *et al*, 2007) indicated significantly increased *DKC1* expression in a subset of prostate cancers with a combination of molecular changes, that is, chromosome 8 alterations and LINE-1 hypomethylation, typical of advanced cases. This prompted us to investigate *DKC1* expression and dyskerin function in prostate cancer.

## Materials and methods

### Tissue samples

Prostate cancer specimens were obtained between 1997 and 2002 by radical prostatectomy as described (Schulz *et al*, 2007). TNM classification was performed according to the guidelines of the International Union Against Cancer from 1997. Of 47 prostate carcinoma tissues used for molecular analyses, 20 carcinomas were staged as pT2, 25 as pT3, and 2 as pT4. Lymph node metastases were present in 12 patients. None of the patients had detectable distant metastases at the time of surgery. Gleason sums of tumours were ⩽6 in 13 cases, 7 in 26 cases, and >7 in 8 cases. The mean patient age was 67 years, ranging from 59 to 74 years. All patients were followed up for a mean period of more than 5 years. During this time, 13 patients suffered recurrences. The study was approved by the ethics committee of the Heinrich Heine University medical faculty.

### Cell lines and cultivation

The prostate carcinoma cell lines, 22Rv1, LNCaP, PC3, and Du145, were cultured in RPMI-1640 (Gibco Life Technologies, Karlsruhe, Germany), supplemented with 10% foetal calf serum (FCS) and 100 U ml^–1^ penicillin/100 *μ*g ml^−1^ streptomycin. MDAPCa2b was cultured in F12K medium supplemented with 20% FCS, 100 *μ*g ml^−1^ penicillin/streptomycin, 25 ng ml^−1^ cholera toxin, 10 ng ml^−1^ epidermal growth factor (Gibco), 5 pM phosphoethanolamine, 10 pg ml^−1^ hydrocortisone (Sigma, Taufkirchen, Germany), and Insulin-Transferrin-Selenium (Gibco). Normal prostate epithelial cells (PrECs) were cultured in Clonetics PrEGM basal medium with supplements recommended by the supplier (Lonza, Verviers, Belgium).

### RNA preparation and quantitative RT–PCR

Total mRNA was isolated from cell cultures grown to 80% confluence by the RNeasy Mini Kit (Qiagen, Hilden, Germany). For tissues, the same kit was used following guanidinium/acid phenol/chloroform extraction (peqGOLD TriFast, PeqLab, Erlangen, Germany). After photometric quantification, 2 *μ*g mRNA was transcribed into first-strand cDNA using SuperscriptII (Invitrogen, Karlsruhe, Germany) with oligo-dT primers according to the manufacturer's protocol. Real-time PCR assays were carried out by commercially available validated primer assays according to the manufacturer's protocol (Applied Biosystems, Weiterstadt, Germany) using the ABI Prism 7900HT real-time PCR system. To assess hTR levels, random primers were used in the reverse transcription procedure and RT–PCR was conducted with the primers 5′-TTCAGCGGGCGGAAAAG-3′, 5′-ACTCGCTCCGTTCCTCTTCCT-3′, and the labelled probe 5′-6FAM-CCTTCCACCGTTCATTCTAGAGCAAACAA-TMR-3′ (TIB MOLBIOL, Berlin, Germany). Expression values for specific genes were related to those for *TBP*, the most stable housekeeping gene during prostate carcinogenesis (Schmidt *et al*, 2006).

### *DKC1* knockdown using RNA interference

Prostate cancer cell lines, 22Rv1 and Du145, were grown in standard conditions and transfected using validated siRNA specific for *DKC1* mRNA (Invitrogen) at 20 nM with Lipofectamine RNAiMAX. A general nontargeting siRNA (Qiagen) was used as a control at the same final concentration.

### Viability, apoptosis, and senescence assays

Cell numbers were determined by the CellTiter-Glo luminescent cell viability assay (Promega, Mannheim, Germany). Apoptosis was followed using the Caspase-Glo 3/7 assay by the same company according to the manufacturer's instructions. For senescence-associated *β*-galactosidase (SA-*β*-Gal) staining, cells were washed in phosphate-buffered saline (PBS) and fixed in 3% formaldehyde at room temperature for 3 min. Staining for enzyme activity was then performed by a standard procedure (Dimri *et al*, 1995) with LNCaP cells induced to become senescent by downregulation of the Snail transcription factor as a positive control.

### DNA extraction and gene copy number determination

High-molecular-weight genomic DNA from tissue and cell lines was isolated using the blood and cell culture DNA kit (Qiagen) as described. Commercially available SNP genotyping assays (Applied Biosystems) were adapted for use in quantitative real-time PCR to measure gene copy numbers in 45 prostate cancer tissues as described (Wlazlinski *et al*, 2007). For *DKC1*, SNP rs3752356 was selected, of which essentially one allele is present in the German population. *TOP2B* (rs11129202) at chromosome 3p was used as a reference gene; two cases with low values in the *TOP2B* measurement, likely because of allele loss, were excluded. Duplicate analyses were carried out for each gene and sample using 10 ng of genomic DNA in an ABI Prism 7300 instrument with detection of FAM and VIC fluorescence labels. The ΔC_t_ method was used for calculation of relative dosage. The average values from four benign prostate tissue DNA samples in each experiment were used as a standard and set as a gene dosage of 2 for *TOP2B* and 1 for *DKC1*.

### Western blot analysis

Total cellular protein extracts in RIPA buffer (5 *μ*g) or nuclear proteins prepared using a commercially available nuclear extraction kit (Active Motif, Carlsbad, CA, USA) were electrophoresed through a 10% SDS–polyacrylamide gel and electroblotted onto immobilon-P membranes (Millipore, Schwalbach, Germany). Nonspecific binding was blocked by incubating the membrane in 5% non-fat milk powder in PBS. Thereafter, membranes were incubated overnight with rabbit polyclonal anti-dyskerin antibody (1 : 3000, Atlas Antibodies, Stockholm, Sweden) and mouse monoclonal anti-TBP (1 : 2000; Abcam, Cambridge, UK) as a loading control at 4°C. After washing, blots were incubated with HRP-conjugated anti-rabbit or anti-mouse secondary antibody (1 : 100 000, Santa Cruz, Heidelberg, Germany) for 1 h, followed by chemoluminescence detection using the ECL advanced kit and exposure to Hyperfilms (Amersham-Pharmacia, Munich, Germany).

### Immunohistochemistry

Immunohistochemical detection of dyskerin in paraffin-embedded tissues was established using biopsy specimens of human cervix uteri as a positive control. To analyse the expression in human prostate tissues, a tissue microarray comprising 24 prostate tissue cores (12 carcinomas, 12 benign prostate tissue samples), as well as three tissue cores of normal placenta, normal lymph node, and normal thyroid gland, respectively, was generated (core diameters: 2 mm each). Paraffin sections were treated with xylene and ethanol. Sections were rehydrated and endogenous peroxidase activity was eliminated by H_2_O_2_. After antigen retrieval, using EDTA (pH 9) in a pressure cooker, endogenous biotin was blocked. Slides were incubated with the dyskerin-specific antibody at a 1 : 50 dilution for 1 h at room temperature and subsequently subjected to the EnVision detection system (Dako, Hamburg, Germany). Reaction products were visualised by immersing slides in diaminobenzidine tetrachloride.

### Statistical methods

Statistical calculations were carried out using SPSS 12.0 (SPSS Inc., Munich, Germany).

## Results

**Figure 1:**
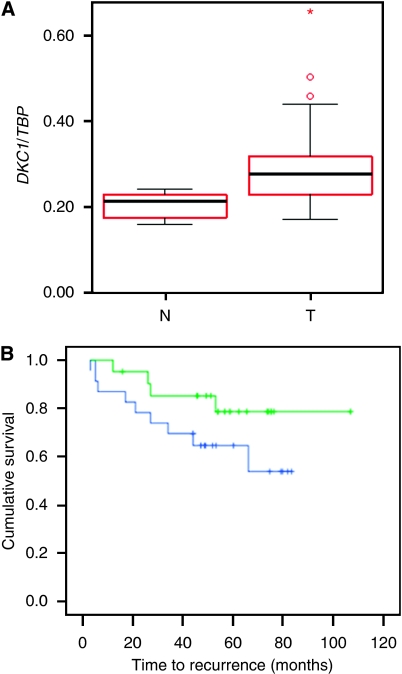
*DKC1* expression in prostate cancers. (**A**) RT–PCR quantitation of expression in prostate carcinoma (T) and benign (N) tissues. (**B**) Kaplan–Meier analysis of the effect of *DKC1* expression on prostate cancer biochemical recurrence. Top curve: below median expression; bottom curve: above median expression.

Expression of *DKC1* was first studied by real-time RT–PCR in a series of 47 M0 prostate carcinomas and 13 benign prostate tissues from prostatectomies (Figure 1A). In the carcinoma tissues, *DKC1* mRNA was highly significantly elevated (*t*-test: *P*<0.001) compared with benign tissues. The increase was significantly stronger in higher-stage cancers (*t*-test: *P*=0.031) and in cases with lymph node metastases (*t*-test: *P*=0.019). Moreover, expression of *DKC1* was significantly elevated in cases with recurrences (*t*-test: *P*=0.030). Kaplan–Meier analysis revealed a marked, but not significant (log-rank: *P*=0.140), difference in the clinical course between tumours, with *DKC1* expression above *vs* below median (Figure 1B).

**Figure 2:**
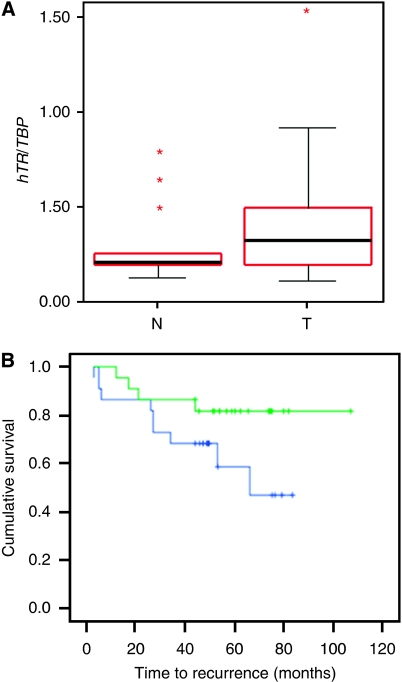
*hTR* expression in prostate cancers. (**A**) RT–PCR quantitation of *hTR* expression in prostate carcinoma (T) and benign (N) tissues. (**B**) Kaplan–Meier analysis of the effect of *hTR* expression on prostate cancer recurrence. Top curve: below median expression; bottom curve: above median expression.

The expression of the RNA subunit of telomerase, hTR, was similarly, on an average, higher in cancer tissues than in benign samples (Figure 2A), but the difference did not reach statistical significance (*t*-test: *P*=0.255). Cases with higher hTR expression tended towards earlier recurrence (Figure 2B), but the difference was not statistically significant according to Kaplan–Meier analysis (log-rank: *P*=0.094). In line with previous reports (Bettendorf *et al*, 2003; Kamradt *et al*, 2003), no associations with histopathological parameters were observed. Expressions of *DKC1* mRNA and hTR were moderately well correlated with each other (Spearman's *ρ*: 0.360, *P*=0.013).

To determine whether increased *DKC1* expression reflects increased tumour proliferation, its relationship to the expression of the proliferation markers *MKI67* (encoding the Ki67 protein) and *PCNA* was determined. A moderate and significant correlation was observed between *DKC1* and *MKI67* mRNA levels (Spearman's *ρ*: 0.353, *P*=0.015), but none with *PCNA* mRNA. Thus, differential levels of *DKC1* mRNA in prostate cancer tissues are related to differences in cell proliferation, but not closely so.

As the *DKC1* gene is located at Xq28 and gain of X-chromosome sequences is often observed in advanced stage prostate cancers, the gene copy number was investigated by quantitative PCR in 45 cancer specimens (see Materials and Methods). Of these, 43 yielded gene copy numbers of 0.4–1.5, with the average of normal tissues set as 1. Two samples showed copy numbers between 2 and 2.5. No significant correlation was observed between *DKC1* gene copy numbers and mRNA expression (Pearson's *r*=0.125). Thus, in primary prostate cancers, gain of Xq28 does not seem to represent a major factor responsible for *DKC1* overexpression.

**Figure 3:**
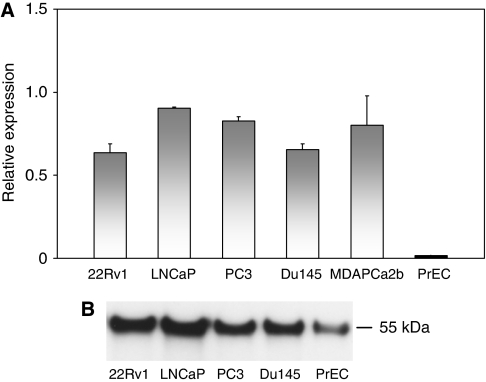
Expression of *DKC1* in prostate cancer cell lines. (**A**) Expression of *DKC1* relative to the reference gene *TBP* in the indicated prostate cancer cell lines and normal prostate epithelial cells (PrECs), according to quantitative RT–PCR. *DKC1* and *TBP* were each measured in triplicate, s.e.m. are indicated. (**B**) Western blot analysis of the dyskerin protein in nuclear extracts of the indicated prostate cell lines and normal PrECs. Equal amounts of nuclear extract from each cell line were loaded and analysed for dyskerin as described in Materials and Methods.

**Figure 4:**
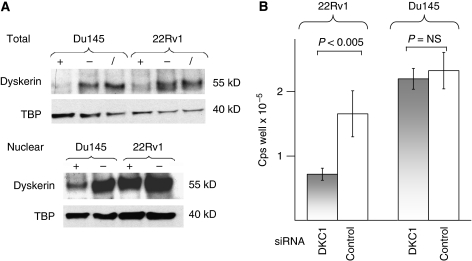
Effect of *DKC1* knockdown in prostate cancer cell lines. (**A**) Western blot analysis of dyskerin expression in Du145 and 22Rv1 after siRNA treatment. Top: total cell extracts; bottom: nuclear extracts; + and − symbols denote cells transfected with DKC1 siRNA and nontargeting siRNA, respectively; the slash symbol denotes untransfected cells. (**B**) Effect of siRNA on ATP amounts as an indicator of vital cell numbers after 3 days of treatment with siRNA directed against DKC1 or nontargeting siRNA (control). *P*-values were determined by *t*-test.

To investigate possible functional consequences of *DKC1* overexpression, we used a siRNA-based approach in prostate cancer cell lines. In cancer cells, as in tissues, *DKC1* mRNA and protein expression surpassed that in cultured normal PrECs (Figure 3). Treatment of 22Rv1 and Du145 prostate carcinoma cell lines with either one of three previously published *DKC1* siRNAs (Montanaro *et al*, 2006) for 3 days diminished *DKC1* mRNA levels by 90–95% compared with cells treated with a control siRNA. At this time point, a strong loss of the protein was observed in Du145 cells, whereas the protein level decreased only moderately in the 22Rv1 cell line (Figure 4). It is noteworthy that the antibody used in these experiments reacted with a number of apparently unrelated proteins in total cell extracts, whereas with nuclear extracts, essentially only a single strong protein band was apparent.

**Figure 5:**
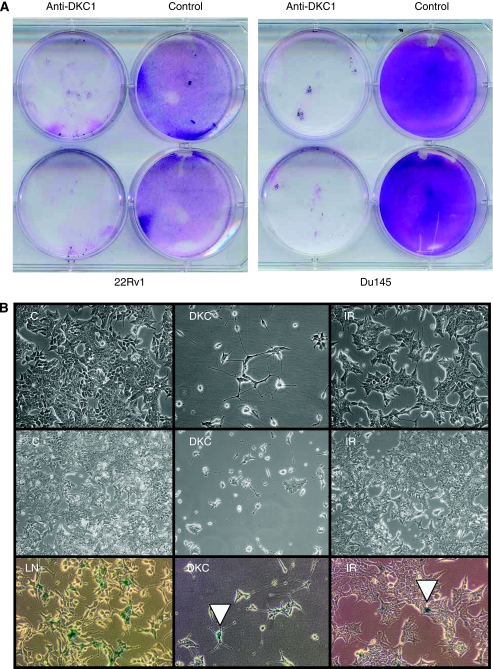
Effect of sustained *DKC1* siRNA treatment on cell proliferation and survival. (**A**) Giemsa staining of culture dishes of the indicated cell lines after 10 days of treatment with siRNA directed against DKC1 or a nontargeting siRNA (control). (**B**) Morphology of 22Rv1 cells after treatment with *DKC1* siRNA (DKC), control siRNA (IR), or mock treated (C) after 6 days (top row) or 10 days (centre row). Bottom row: results of staining for SA-*β*-Gal activity in 22Rv1 cells treated for 6 days with *DKC1* siRNA (DKC) or control siRNA (IR). Individual cells staining positively under either condition are indicated by arrowheads in both panels. LN+ is a positive control of LNCaP cells induced to senescence by downregulation of the Snail transcription factor (WA Schulz *et al*, unpublished observation).

After 3 days of treatment with siRNA directed against *DKC1*, cell numbers – as measured by overall ATP levels – were moderately decreased in the 22Rv1 line, but not significantly in Du145 cells (Figure 4B). At this time point, no significant increase in apoptosis was observed. An assay for active caspase 3/7 yielded values of 3877±211 and 6086±1178 for 22Rv1 cells treated with *DKC1* siRNA and control siRNA, respectively, and of 1239±110 and 1310±134, respectively, for Du145 cells. A more dramatic effect was exerted by long-term *DKC1* knockdown. Du145 or 22Rv1 cells were treated with specific *DKC1* siRNA or with control siRNA for 3 days and then passaged at the usual ratio. After 1 day of recovery, siRNA treatment was repeated and cells were passaged again 3 days later. After the third passage (that is, 10 days in all), cells treated with control siRNA were still proliferating and the cell density was only slightly lower than that in untreated cells, whereas only a few cells treated with siRNA against *DKC1* survived (Figure 5). Notably, cells treated with *DKC1* siRNA neither exhibited morphological signs of senescence nor stained positively for the senescence marker, SA-*β*-Gal (Figure 5B). Instead, the main effect on 22Rv1 cells was that they became diminutive, with an elongated shape and often several thin extensions, and reattached poorly to the cell culture dish after passaging (Figure 5B). In the Du145 line, lack of adhesion was the major factor leading to cell loss. Also, after 10 d treatment, occasional apoptotic cells became morphologically evident in this cell line. However, the cell numbers at that time were too low for quantitative determination of the extent of apoptosis.

**Figure 6:**
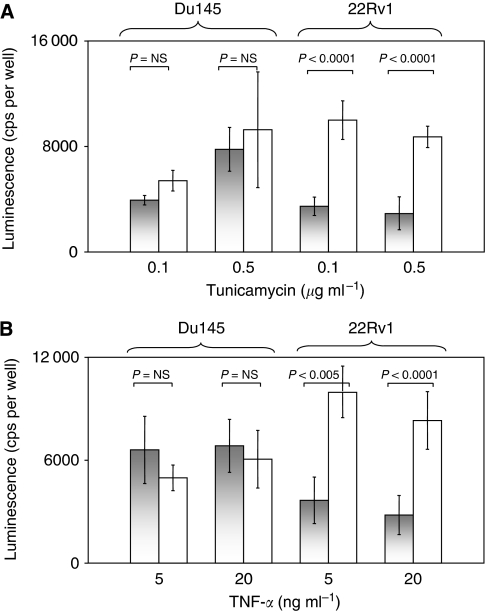
Effect of *DKC1* knockdown on induction of apoptosis by tunicamycin or TNF-*α*: (**A**) tunicamycin, (**B**) TNF-*α*. In each panel, apoptosis was followed up using a luminescence caspase 3/7 activity assay. Grey bars: treatment with *DKC1* siRNA, white bars: treatment with nontargeting siRNA. *P*-values were determined by *t*-test.

To investigate the reported anti-apoptotic effect of dyskerin (Yoon *et al*, 2006) in prostate cancer cells, Du145 or 22Rv1 cells pretreated with siRNA for 3 days were challenged with apoptosis inducers TNF-*α* or tunicamycin (Shiraishi *et al*, 2005). The particular proapoptotic agents were chosen, because death receptor ligands such as TNF-*α* and endoplasmic reticulum stress, through which tunicamycin acts, are thought to be particularly important in prostate cancer. When caspase 3/7 activity was determined as an indicator of apoptosis 24 h later, no significant differences were observed between Du145 cells treated with *DKC1* siRNA or with a nontargeting control siRNA, whereas caspase activity was rather diminished than enhanced by *DKC1* knockdown in 22Rv1 cells treated with either inducer of apoptosis (Figure 6) mirroring the findings in cells not treated with a proapoptotic agent described above.

To verify that increased *DKC1* mRNA expression in prostate cancer results in increased protein levels, immunohistochemical analyses for dyskerin were carried out on paraffin-embedded tissues (Supplementary Figure 1). Optimal staining conditions were established using biopsy specimens of human cervix uteri as a positive control. In these, dyskerin was found to be strongly expressed in nucleoli of both squamous and cylindrical epithelial cells, although in a focal manner. Next, we investigated dyskerin expression in 24 prostate tissue samples on a tissue microarray with 12 matched tissue cores of prostate cancer and the respective normal counterparts. We observed unspecific moderate-to-strong immunohistochemical staining in the cytoplasm of both cancerous and normal prostatic glands, but could not detect a specific nucleolar dyskerin expression. To exclude heterogeneous expression in tissues as a source of this failure, dyskerin expression was further investigated in large prostate cancer tissue sections from two patients. Indeed, in one of these samples, we found a few tumour cells with a strong nucleolar dyskerin expression, whereas the vast majority of tumour cells, as well as all other cells, remained negative. From these observations we conclude that the commercially available antibody is not sensitive enough to allow a systematic analysis of dyskerin expression in paraffin-embedded prostatic tissues.

## Discussion

Our data indicate that *DKC1* overexpression is common in prostate cancer, and is associated with adverse histopathological features, especially tumour extension. These data fit excellently with those reported by others for breast cancer (Montanaro *et al*, 2006, 2008a), suggesting that overexpression of *DKC1* might be a frequent property of aggressive carcinomas. It is noteworthy that we observed only a weak-to-moderate association between *DKC1* levels and actual markers of tumour proliferation, such as *MKI67* and *PCNA*, suggesting that the increase in *DKC1* expression is not simply a by-product of enhanced cell proliferation.

Dyskerin functions in several cellular processes, such as pseudouridine formation and consequently RNA and general protein biosynthesis (Filipowicz and Pogacic, 2002), biosynthesis of hTR, and stabilisation of the telomerase complex (Chang *et al*, 2002; Cohen *et al*, 2007), as well as regulation of apoptosis (Yoon *et al*, 2006). Downregulation of dyskerin by siRNA in prostate cancer cells did not elicit apoptosis by itself or sensitise to induction of apoptosis by TNF-*α* or tunicamycin, agents chosen because of their relevance to prostate cancer (Shiraishi *et al*, 2005). In fact, in the 22Rv1 cell line, activation of caspase 3/7 by these agents seemed rather muted by the downregulation of dyskerin. As growth of 22Rv1 was more rapidly affected by *DKC1* knockdown than that of Du145, it is possible that this surprising effect may be a consequence of already diminished protein biosynthetic capacity in this cell line.

Neither were morphological signs of senescence nor increased expression of the senescence marker SA-*β*-Gal induced by *DKC1* siRNA, which would be expected if loss of telomerase function was the primary effect of dyskerin downregulation in these cells. Complete cessation of cell growth and proliferation was observed after at most three passages, corresponding to 10 days of treatment. By comparison, inhibition of hTERT by a dominant-negative protein arrests the proliferation of prostate cancer cell lines only after several weeks (Guo *et al*, 2001).

Thus, the most critical function of dyskerin in prostate cancer cells seems to lie in its role in protein biosynthesis. The phenotype elicited by *DKC1* siRNA, that is, a gradual cessation of proliferation, decreased cell size, and inability to reattach after trypsinisation or even spontaneous detachment, is fully compatible with a defect in protein biosynthesis. In keeping with this interpretation, in breast cancer cells, a close correlation between pseudouridine levels and *DKC1* expression has been directly demonstrated (Montanaro *et al*, 2006). Therefore, in breast as well as prostate cancers, overexpression of dyskerin may be a primary necessity to support the increased RNA and protein biosynthesis of cancer cells. In addition, the observation that *DKC1* mRNA expression in prostate cancer tissues correlated, if only moderately, with hTR levels supports the idea that for the long-term growth of tumours *in vivo*, dyskerin may additionally be important to maintain telomerase activity. The same conclusion has recently been reached for breast cancers (Montanaro *et al*, 2008a).

It remains to be demonstrated that the increased levels of *DKC1* mRNA in prostate cancer tissues result in an overexpression of the protein, as was shown for breast cancer (Montanaro *et al*, 2006). Moreover, in view of the association of increased *DKC1* mRNA with prostate cancer stage and a tendency towards association with recurrence, it seems highly desirable to explore the prognostic value of dyskerin expression using tissue microarrays. Unfortunately, we could not perform a systematic analysis in paraffin-embedded benign or cancerous prostate cancer tissues from archival samples, as the commercial polyclonal antibody successfully used in western blotting with nuclear extracts (Figures 3 and 4) detected nucleolar dyskerin expression in a subset of normal uterine cervical cells, but only in very few prostate cancer cells, and the polyclonal antibody used on breast cancers (Montanaro *et al*, 2006) is depleted. Therefore, this analysis has to be postponed until an appropriate antibody is available.

At first glance, the finding that *DKC1* is overexpressed in common carcinomas seems somewhat paradoxical, given that dyskerin-inactivating mutations in dyskeratosis congenita predispose to cancer development, and *DKC1* is downregulated in sporadic CLL (Poncet *et al*, 2008). Moreover, a complete lack of dyskerin function is lethal. It seems, though, that the mutant proteins in dyskeratosis congenita retain pseudouridine synthase activity, but by failing to stabilise telomerase, impair long-term cell proliferation and elicit activation of checkpoints (Gu *et al*, 2008). In these circumstances, cells with mutations allowing proliferation in the absence of functional telomeres and regardless of checkpoint activation would possess a huge selective advantage, which ultimately leads to cancer. According to this argument, in tumours arising in dyskeratosis congenita, dyskerin mutations would constitute the primary event and favour the development of secondary changes that initiate cancer development. Conversely, in the progression of breast and prostate carcinomas, dyskerin overexpression may represent a secondary change necessary to meet the requirements for increased RNA biosynthesis and telomerase activity.

The precise mechanisms leading to overexpression of dyskerin in common carcinomas remain to be elucidated in further studies. As we did not observe increased gene copy numbers in prostate cancers with *DKC1* overexpression, they are likely epigenetic. In this respect, it could be significant that *DKC1* was recently identified as a direct target of MYC (Alawi and Lee, 2007), a major regulator of cancer cell growth frequently overexpressed in aggressive breast and prostate cancers.
