# Supplementary figures and images for: DKC1 overexpression associated with prostate cancer progression
Source: Br J Cancer. 2009 Sep 15;101(8):1410–6. doi: 10.1038/sj.bjc.6605299 (PMC2768451; doi:10.1038/sj.bjc.6605299)

## Slide 1
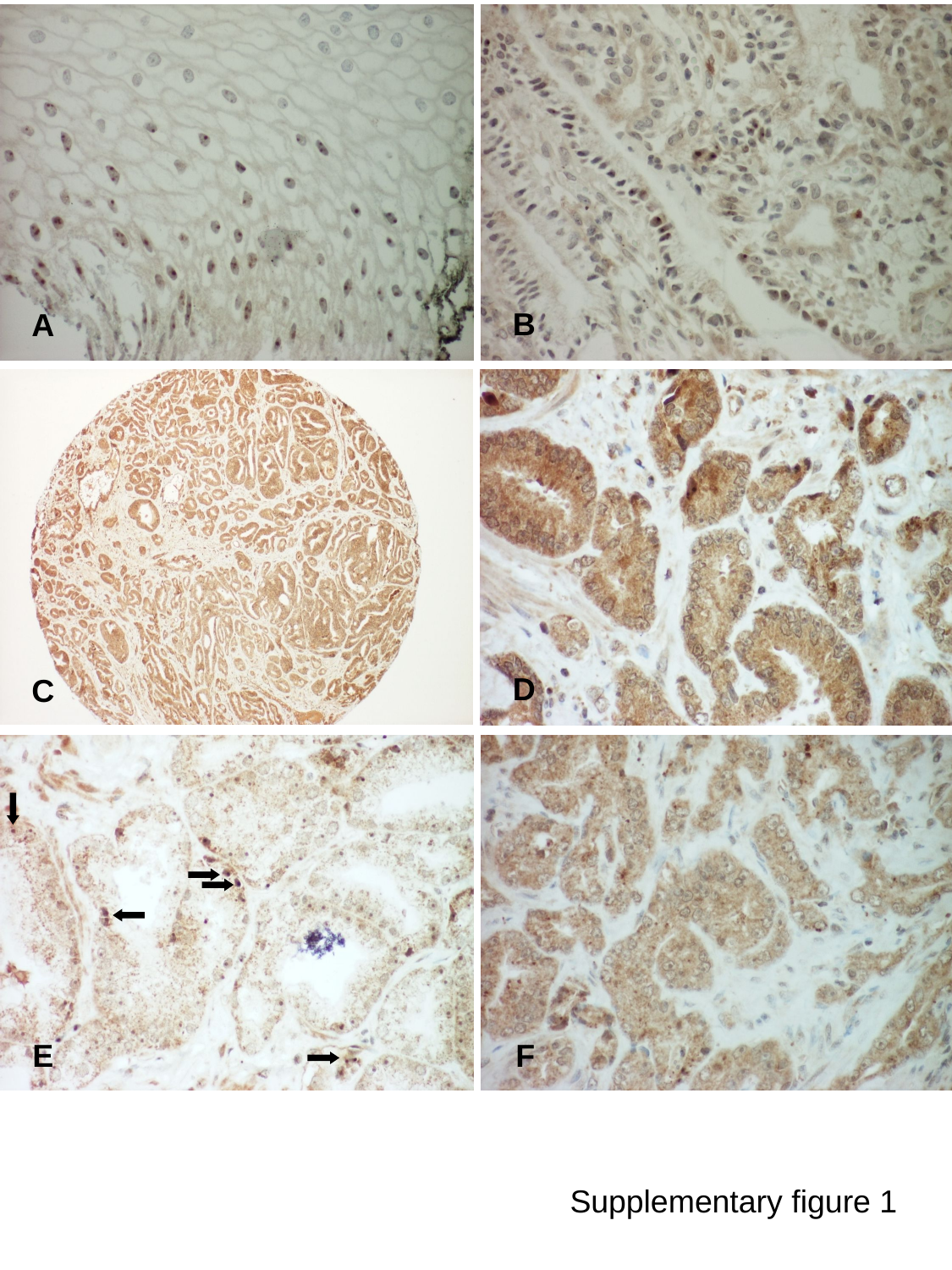

B
A
D
C
E
F
Supplementary figure 1

Supplement: Supplementary Figure 1 [file 6605299x1.ppt]
