# Supplementary material for: DKC1 overexpression associated with prostate cancer progression
Source: Br J Cancer. 2009 Sep 15;101(8):1410–6. doi: 10.1038/sj.bjc.6605299 (PMC2768451; doi:10.1038/sj.bjc.6605299)
Supplement: Supplementary Figure 1 Legend [file 6605299x2.doc]

supplementary Fig. 1: Immunohistochemical analysis of Dyskerin expression in biopsy specimens of the cervix uteri and in prostate carcinomas. (A and B) Heterogeneous nucleolar DKC1 expression of squamous (A) and cylindric (B) epithelium of the cervix uteri. (C and D) Representative stainings for Dyskerin on a prostate tissue microarray at low (C) and high (D) magnification. Except for an unspecific cytoplasmic reaction no specific nucleolar positivity was observed in any of the tissue cores investigated. (E and F) Analysis of large prostate cancer tissue sections showing nucleolar Dyskerin expression in only a few tumor cells (E, arrows), whereas the majority of tumor cells exhibit an unspecific diffuse and dot-like cytoplasmic reaction (F).
